# Skeletal Muscle Insulin Resistance Associated with Cholesterol-Induced Activation of Macrophages Is Prevented by High Density Lipoprotein

**DOI:** 10.1371/journal.pone.0056601

**Published:** 2013-02-21

**Authors:** Andrew L. Carey, Andrew L. Siebel, Medini Reddy-Luthmoodoo, Alaina K. Natoli, Wilissa D’Souza, Peter J. Meikle, Dmitri Sviridov, Brian G. Drew, Bronwyn A. Kingwell

**Affiliations:** 1 Metabolic and Vascular Physiology Laboratory, Baker IDI Heart and Diabetes Research Institute, Melbourne, Victoria, Australia; 2 Lipoproteins and Atherosclerosis Laboratory, Baker IDI Heart and Diabetes Research Institute, Melbourne, Victoria, Australia; 3 Metabolomics Laboratory, Baker IDI Heart and Diabetes Research Institute, Melbourne, Victoria, Australia; 4 Division of Endocrinology, Diabetes and Hypertension, University of California Los Angeles, Los Angeles, California, United States of America; Tohoku University, Japan

## Abstract

**Background:**

Emerging evidence suggests that high density lipoprotein (HDL) may modulate glucose metabolism through multiple mechanisms including pancreatic insulin secretion as well as insulin-independent glucose uptake into muscle. We hypothesized that HDL may also increase skeletal muscle insulin sensitivity via cholesterol removal and anti-inflammatory actions in macrophages associated with excess adiposity and ectopic lipid deposition.

**Methods:**

Human primary and THP-1 macrophages were treated with vehicle (PBS) or acetylated low density lipoprotein (acLDL) with or without HDL for 18 hours. Treatments were then removed, and macrophages were incubated with fresh media for 4 hours. This conditioned media was then applied to primary human skeletal myotubes derived from vastus lateralis biopsies taken from patients with type 2 diabetes to examine insulin-stimulated glucose uptake.

**Results:**

Conditioned media from acLDL-treated primary and THP-1 macrophages reduced insulin-stimulated glucose uptake in primary human skeletal myotubes compared with vehicle (primary macrophages, 168±21% of basal uptake to 104±19%; THP-1 macrophages, 142±8% of basal uptake to 108±6%; P<0.05). This was restored by co-treatment of macrophages with HDL. While acLDL increased total intracellular cholesterol content, phosphorylation of c-jun N-terminal kinase and secretion of pro- and anti-inflammatory cytokines from macrophages, none were altered by co-incubation with HDL. Insulin-stimulated Akt phosphorylation in human skeletal myotubes exposed to conditioned media was unaltered by either treatment condition.

**Conclusion:**

Inhibition of insulin-stimulated glucose uptake in primary human skeletal myotubes by conditioned media from macrophages pre-incubated with acLDL was restored by co-treatment with HDL. However, these actions were not linked to modulation of common pro- or anti-inflammatory mediators or insulin signaling via Akt.

## Introduction

While circulating high-density lipoprotein (HDL) is well-established to protect against atherosclerotic vascular disease, there is increasing evidence that it may also have anti-diabetic properties [1]–[4]. The mechanisms accounting for the actions of HDL on glucose metabolism are multiple and include actions both in the pancreas [2], [4] and skeletal muscle [2]. In the pancreas, HDL promotes glucose-stimulated insulin secretion via both direct effects [4] as well as secondary to cholesterol efflux [5]–[8]. HDL also acts directly on skeletal muscle to enhance insulin-independent glucose uptake through direct signaling pathways involving AMPK [2], but recent evidence suggests an additional effect of HDL on insulin-dependent pathways [1]. In the Investigation of Lipid Level Management to Understand its Impact in Atherosclerotic Events (ILLUMINATE) trial, HDL-elevation with the cholesteryl ester transfer protein (CETP) inhibitor, torcetrapib reduced plasma glucose, insulin and HOMA-IR in patients with diabetes, suggesting that the treatment may have improved insulin sensitivity. It is possible that effects of HDL on insulin sensitivity may be secondary to the anti-inflammatory actions of HDL on activated immune cells [9], [10].

In the context of atherosclerosis, HDL inhibits inflammatory events via both direct transcriptional mechanisms and via cellular cholesterol removal [11], [12]. The anti-inflammatory actions of HDL on macrophages may also have relevance in mitigating against insulin resistance associated with macrophage inflammatory responses. Obesity is associated with macrophage infiltration and activation in adipose tissue [13]. Consequent cytokine release, particularly tumour necrosis factor (TNF)-α, impairs insulin signaling and sensitivity in skeletal muscle [13]–[15]. The reduction in plasma HDL concentration and functionality associated with obesity and type 2 diabetes [16], [17], may therefore be linked to impaired insulin sensitivity in skeletal muscle, which accounts for the majority of insulin-stimulated glucose disposal in the body [18]. We hypothesized that HDL increases insulin-sensitivity via cholesterol removal and anti-inflammatory actions in macrophages associated with excess adiposity/ectopic lipid deposition. In this study we used primary myotube cultures derived from newly diagnosed, unmedicated, patients with early-stage type 2 diabetes, since this is a population for whom HDL elevation may be clinically relevant. The specific aims were to determine whether:

- Addition of conditioned media from acetylated low density lipoprotein (acLDL) loaded human macrophages (primary and THP-1) to myotubes would impair insulin-stimulated glucose uptake.
- Co-treatment of cholesterol-loaded macrophages with HDL would ameliorate the effects of acLDL on insulin-stimulated skeletal muscle glucose uptake in myotube cultures from patients with type 2 diabetes.
- The effect of HDL could be attributed to cholesterol removal and anti-inflammatory actions on macrophages.

Inhibition of insulin-stimulated glucose uptake in primary human skeletal myotubes by conditioned media from macrophages pre-incubated with acLDL was restored by co-treatment with HDL. However, these actions were not linked to modulation of common pro- or anti-inflammatory mediators or insulin signaling via Akt. This study together with a growing body of evidence linking HDL to glucose metabolism, may inform development of therapeutics aimed at elevating plasma HDL levels in the context of type 2 diabetes.

## Methods

### Study Design Overview

Human primary and THP-1 macrophages were incubated with vehicle (PBS), acLDL with or without HDL for 18 hours. Treatments were then removed, and macrophages were incubated with fresh media. After 4 hours, conditioned media, from both human primary and THP-1 macrophages was applied to primary human skeletal myotubes derived from vastus lateralis biopsies from patients with type 2 diabetes at a final concentration of 2% (diluted in regular media). After 24 hours insulin-stimulated (30 min) glucose uptake and Akt phosphorylation were examined. Cytokines and phosphorylation of the hierarchical regulator of macrophage inflammation JNK were also examined in the conditioned media as well as cholesterol content of macrophages.

This study was approved by the Alfred Hospital Human Research Ethics Committee, and informed written consent was obtained from all volunteers.

### Primary Human Monocyte Culture

Fourteen healthy volunteers (31.6±2.1 yrs; weight 69.5±2.5 kg; BMI 23.6±0.7 kg/m^2^; fasting glucose 4.9±0.1 mmol/l; mean ± SEM) each donated a total of 150 ml of blood.

Human monocytes were isolated by density-gradient centrifugation (Ficoll-Paque; GE Healthcare, Rydalmere, NSW, Australia), followed by Miltenyi MACS magnetic bead sorting for CD14, as per manufacturer’s instructions (Miltenyi Biotech, North Ryde, NSW, Australia). In preliminary experiments population purity of monocytes utilizing this protocol were analyzed and found to be >80%, as determined by immunofluorescent staining with anti-CD14 monoclonal antibody (BD Biosciences, North Ryde, NSW, Australia) and forward and side scatter analysis (FACSCalibur; BD Biosciences, North Ryde, NSW, Australia). Monocytes were resuspended at a concentration of 1×10^6^ cells/ml in Iscove’s Modified Dulbecco’s Medium (IMDM), supplemented with 10% human serum and 1% penicillin-streptomycin (Invitrogen, Mulgrave, Vic, Australia) and 50 ng/ml macrophage colony stimulating factor (MCSF; Sigma-Aldrich, Castle Hill, NSW, Australia) then plated on extracellular matrix coated 6-well plastic cell culture plates to differentiate for 5 days.

### THP-1 Macrophage Cell Culture

THP-1 macrophages, a human monocyte cell line, were cultured in RPMI 1640 Medium supplemented with 10% heat-inactivated fetal calf serum and 1% penicillin-streptomycin. Cells were seeded in 6-well plates at a density of 2×10^6^/well, and differentiated to macrophages using phorbol-12-myristate-13-acetate (PMA, 100 ng/ml; Sigma), for 3 days.

### Skeletal Muscle Cell Culture

Potential participants were screened for type 2 diabetes using standard criteria (fasting plasma glucose >7.1 mmol/l or a 2 hour blood glucose level of >11.1 mmol/l after a 75 g oral glucose load; oral glucose tolerance test). Five unmedicated, male volunteers with newly identified type 2 diabetes (**Table 1**) underwent a percutaneous vastus lateralis muscle biopsy (∼120 mg) from which satellite myoblasts were isolated [19]. The cells were seeded on extracellular matrix coated plates and cultured to confluence in normal growth media (α-MEM supplemented with 10% fetal calf serum and 1% penicillin-streptomycin) and differentiated to myotubes in α-MEM containing 2% horse serum for 5 days.

**Table 1:** Skeletal muscle biopsy/myocyte culture participant characteristics (n = 5).

Variable	Mean ± SEM
**Physical characteristics**	
** Age (years)**	53±4
** Weight (kg)**	101±9
** BMI (kg/m^2^)**	34±2
** Waist:Hip**	1.0±0.1
**Cardiovascular**	
** Systolic blood pressure (mmHg)**	124±3
** Diastolic blood pressure (mmHg)**	82±5
** Heart rate (bpm)**	64±7
**Fasting hormones, metabolites, lipids**	
** Glucose (mmol/l)**	8.3±2.5
** Insulin (pmol/l)**	98±21
** HbA_1c_ (%)**	8.5±1.0
** HDL cholesterol (mmol/l)**	1.0±0.1
** LDL cholesterol (mmol/l)**	3.0±0.3
** Total cholesterol (mmol/l)**	4.8±0.4
** Triacylglycerol (mmol/l)**	1.7±0.2

BMI, body mass index; HbA_1c_, glycated haemoglobin, HDL; high density lipoprotein; LDL, low density lipoprotein.

### Lipoprotein Isolation and Treatment

HDL and LDL were isolated by isopycnic gradient ultracentrifugation from pooled plasma taken from healthy individuals, as previously described [4] with modification of the density gradient to isolate total HDL (HDL2 and HDL3) from 1.085–1.21 g/ml [20]. To acetylate LDL, 150 µl of saturated sodium acetate and 2 µl of acetic anhydride was added per mg of LDL. After isolation, lipoproteins were dialyzed in phosphate-buffered saline (PBS) containing EDTA for 24 hours and stored at 4°C. HDL and acLDL were both stored at 4°C for up to 8 weeks. Total protein content (BCA protein assay reagents; BioRad, Gladesville, NSW, Australia) of all lipoprotein isolates and their derivatives were quantified and added to cultures in units based on total protein content, according to our previous studies [2]. The lipid loading condition of acLDL included addition of 10 g/ml Sandoz 58-035 reagent (Sigma Aldrich, Castle Hill, NSW, Australia) to inhibit Acyl-CoA: cholesterol acyltransferase (ACAT), resulting in high levels of free intracellular cholesterol, and hereafter will be referred to as acLDL treatment.

### Conditioned Media Experiments

Human primary or THP-1 macrophages were incubated in 2% serum with vehicle (PBS) or with/without HDL (50 µg/ml) containing 75 µg/ml acLDL for 18 hours. They were then incubated with fresh serum-free media+0.1% BSA for 4 hours, which was then collected and used for subsequent experiments at a concentration of 2%.

### Glucose Uptake

Glucose uptake was determined by the well-established 2-deoxy-glucose method [21]. Non-transporter mediated uptake was assessed using cytochalasin-B (10 µmol/L), which was subtracted from total 2-deoxy-glucose uptake.

### Western Blotting

Primary human skeletal myotubes were incubated in α-MEM supplemented with 2% horse serum and treated for 18 hours with conditioned media (described above) prior to insulin treatment for 30 minutes. Cells were washed with ice-cold PBS, lysed in protein lysis buffer and western blotting performed as previously described [2]. Phosphorylation of Akt was measured using an antibody to detect phosphorylation of Serine-473 (Cell Signaling, Danvers, MA, USA). Total beta-actin protein was quantified as an endogenous control protein (Cell Signaling). Immuno-reactive bands were detected using an anti-rabbit HRP-conjugated secondary antibody (BioRad) followed by enhanced chemiluminescence imaging on a BioRad Geldoc XRS+ system (BioRad) and quantified on Quantity One software (BioRad).

THP-1 macrophages were incubated in RPMI with 2% horse serum and treated for designated times with either vehicle (PBS), HDL (50 µg/ml) and/or acLDL (75 µg/ml). Cells were washed in PBS and lysed in protein lysis buffer and western blotting performed as previously described [2]. Phosphorylation of JNK was measured using an antibody to detect phosphorylation of Threonine-183 and Tyrosine-185 (Cell Signaling). Immuno-reactive bands were detected using an anti-rabbit HRP-conjugated secondary antibody (BioRad), visualized and quantified as described above.

### Multiplex Cytokine Assay

Cytokine (granulocyte-monocyte colony-stimulating factor (GM-CSF); interleukin (IL)-8; TNF-α; IL-1 receptor antagonist (IL-1ra); monocyte chemoattractant protein 1 (MCP1); macrophage inflammatory protein (MIP)-1α; MIP-1β; regulated upon activation normal T-cell expressed and secreted (RANTES); IL-10) concentrations were simultaneously quantified in secretion media collected from human primary macrophages treated with vehicle (PBS), acLDL vs. acLDL with HDL for 18 hours using a Human Fluorokine MultiAnalyte Profiling kit (Human MultiAnalyte Profiling Base Kit A, RnD Systems, Minneapolis, MN, USA).

### Intracellular Cholesterol and Protein Content

Intracellular cholesterol and protein content were determined in cell extracts (2×10^6^ cells/ml). Total cholesterol (free cholesterol and cholesteryl esters) was measured using an Amplex Red cholesterol assay kit (Invitrogen). Cells were lysed in phosphate-buffered saline, then assayed according to the manufacturer’s instructions, and corrected for total cell protein using a BCA Protein Assay Reagent (Biorad).

### Statistical Analyses

Data were compared by one-way ANOVA or repeated-measures ANOVA, with least significant difference (LSD) post-hoc tests used to compare individual means as appropriate. Results are expressed as mean ± SEM, and all analyses were conducted using SPSS (v15) software. A significance level of P<0.05 was used.

## Results

### Media From acLDL-loaded Macrophages Impairs Insulin-mediated Skeletal Muscle Glucose Uptake and is Restored by HDL Co-treatment

**Figure 1:**
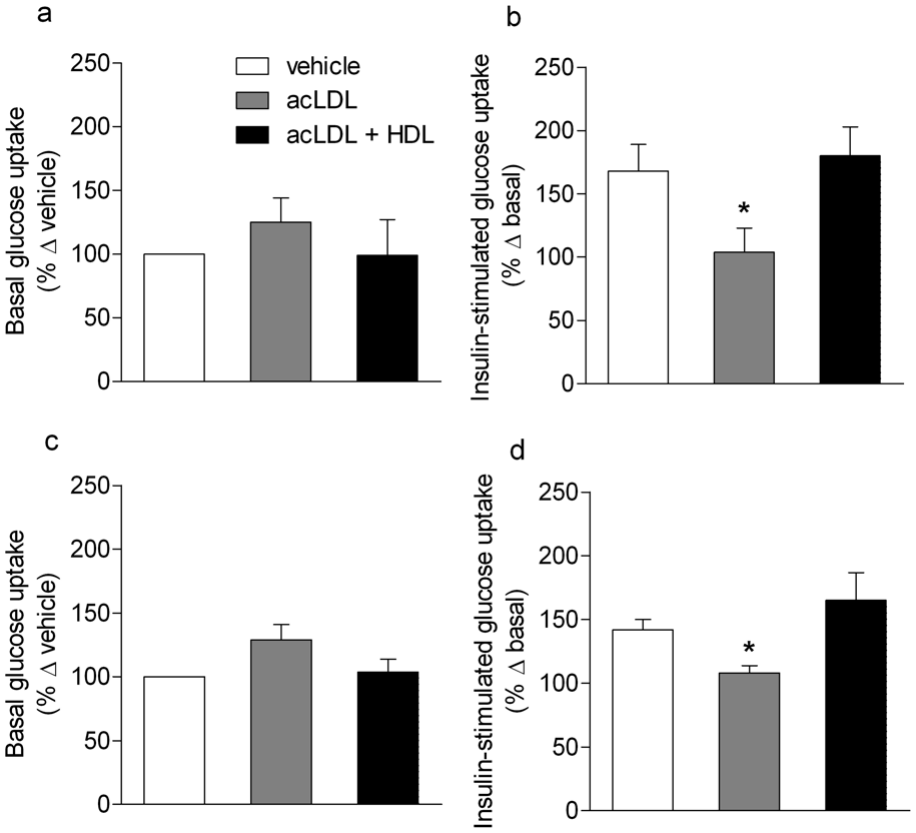
Basal and insulin-stimulated glucose uptake in cultured skeletal myotubes. Conditioned media (2% in final media volume) from **(a,** basal state; **b** insulin-stimulated**)** primary human and **(c,** basal state; **d** insulin-stimulated**)** THP-1 macrophages treated with vehicle (PBS), 75 µg/ml acLDL/10 µg/ml Sandoz compound with or without 50 µg/ml HDL for 18 hours was placed on human primary skeletal myotubes for 24 hours. Cells were treated with insulin (100 nM) or vehicle for 30 minutes before glucose uptake was measured. n = 6/group; data are presented as mean ± SEM; *indicates significantly different from Con and acLDL+HDL, P<0.05.

Basal glucose uptake (in the absence of insulin) in human skeletal muscle myotubes was not different between any of the three conditioned media treatments (vehicle, acLDL or acLDL plus HDL) whether expressed as absolute/raw values or as a percentage change from vehicle (**Figure 1a**). In response to insulin, vehicle-treated, pre-conditioned media elicited a robust increase in glucose uptake of 168±21% (relative to basal of 100%) (**Figure 1b**) which was of a magnitude similar to that observed in the absence of pre-conditioned media (164±20%, n = 6, p = 89). Compared to the vehicle condition, treatment with acLDL decreased insulin-stimulated glucose uptake significantly to 104±19% above basal (P<0.05, **Figure 1b**). Co-treatment of macrophages with HDL and acLDL reversed this effect, increasing insulin-stimulated glucose uptake to 180±23% of basal glucose uptake (P<0.05, **Figure 1b**). This experiment was repeated utilizing THP-1 macrophages, and yielded a similar result. Basal glucose uptake was not different between groups (**Figure 1c**). Insulin-stimulated glucose uptake decreased from 142±8% of basal in the vehicle condition to 108±6% (P<0.05) after incubation with conditioned media from macrophages treated with acLDL. Co-treatment of macrophages with HDL and acLDL again reversed this effect, increasing insulin-stimulated glucose uptake to 165±22% of basal glucose uptake (P<0.05).

### The Effect of HDL is not Associated with Phosphorylation State of Akt in Skeletal Muscle or Reduction in Macrophage Cholesterol Content

**Figure 2:**
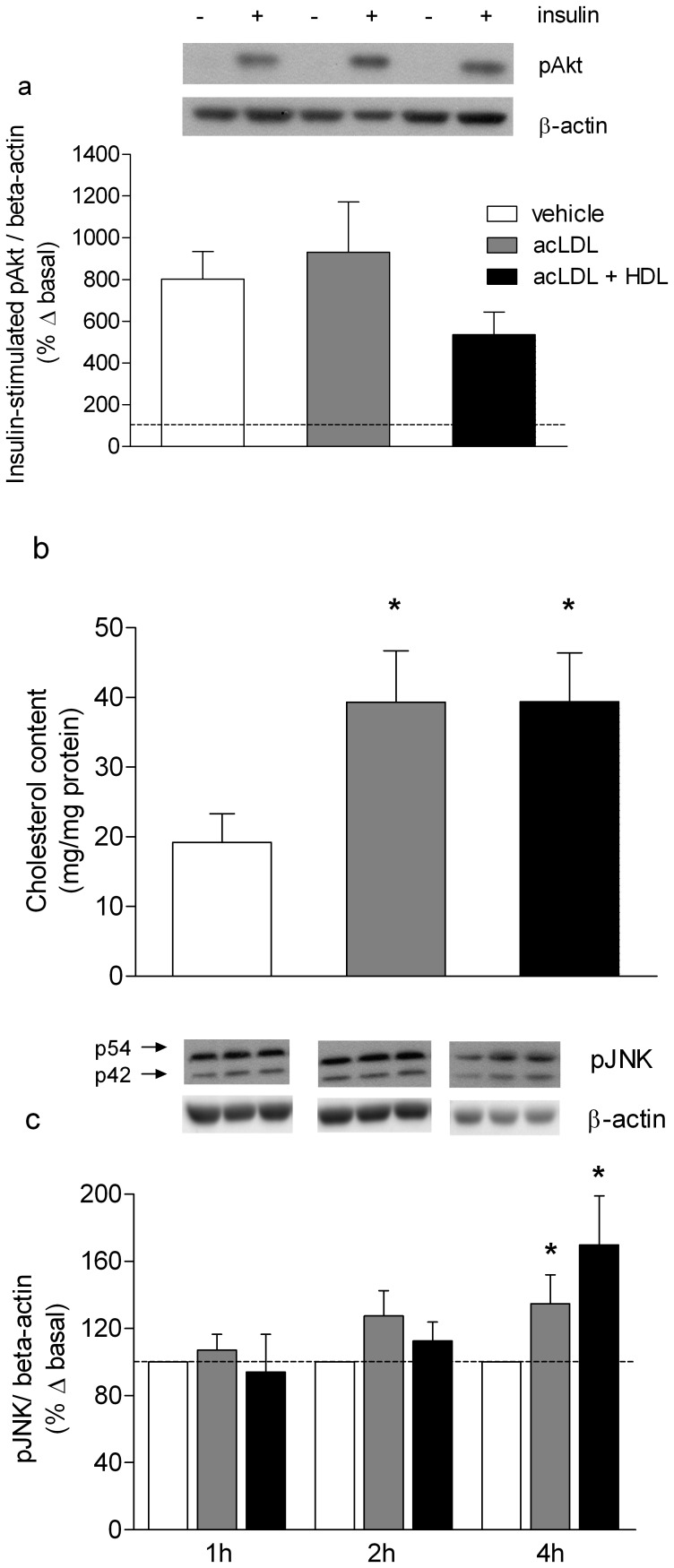
Myotube Akt phosphorylation, human primary macrophage cholesterol content and THP-1 macrophage JNK phosphorylation. (a) Phosphorylation of Akt at Serine-473 in human primary skeletal myotubes treated as for Figure 1, n = 6/group. **(b)** Total intracellular cholesterol (free cholesterol and cholesteryl esters) in human primary macrophages after treatment with vehicle (PBS) or 75 µg/ml acLDL/10 µg/ml Sandoz with or without 50 µg/ml HDL for 18 hours, n = 6/group. **(c)** Phosphorylation of JNK in THP-1 macrophages treated with vehicle or 75 µg/ml acLDL/10 µg/ml Sandoz with or without 50 µg/ml HDL for 1, 2 and 4 hours. n = 9/group; data are presented as mean ± SEM; *indicates significantly different from Con at the corresponding time point, P<0.05.

Human skeletal myotubes were incubated with conditioned media as described for the glucose uptake experiments above. Vehicle-treated pre-conditioned media resulted in a significant and robust (802±132%) phosphorylation of Akt at serine-473, a common marker of insulin-stimulated Akt activity, and this was not affected by lipid loading with acLDL, or co-treatment with HDL (**Figure 2a**). Treatment of human primary macrophages with acLDL increased total intracellular cholesterol content by 104±39% compared to vehicle (P<0.05). Cholesterol content of cells incubated with both acLDL and HDL was similar to that of cells incubated with acLDL alone (**Figure 2b**).

### Treatment of Cholesterol-loaded Macrophages with HDL Neither Reduces Phosphorylation of JNK nor Inflammatory Cytokine Release from Macrophages

Phosphorylation of the hierarchical regulator of macrophage inflammation JNK was unchanged between cells treated with vehicle, acLDL or acLDL plus HDL in THP-1 macrophages after treatment for either 1 or 2 hours in RPMI media containing 2% fetal calf serum (**Figure 2c**). However, after 4 hours phosphorylation of pJNK was increased in cells treated with acLDL alone (135±17%) or in combination with HDL (170±29%; **Figure 2c**, P<0.05). Concentrations of IL-8, TNF-α, MIP-1α and MIP-1β in conditioned media from treated human primary macrophages were elevated by 18 hour treatment with acLDL (P<0.05) and remained elevated with the addition of HDL (**Table 2**). There was no detectable GM-CSF or IL-10 protein measured in the vehicle samples, whereas there were detectable amounts after acLDL treatment with and without HDL (**Table 2**).

**Table 2:** Concentration (pg/ml, mean ± SEM) of cytokines measured in media taken from primary human macrophages after incubation with vehicle (PBS) or acLDL with or without HDL.

Cytokine (pg/ml)	Vehicle	acLDL	acLDL+HDL
**GM-CSF**	ND	483±243	520±241
**IL-8**	2191±1077	62931±22647*	71201±31284*
**TNF-α**	27±10	3847±1055*	2784±994*
**IL-1 RA**	71536±29032	122636±36818	120776±41493
**MCP1**	3126±1319	7725±2989	8837±3736
**MIP-1α**	99±27	56225±16782*	44473±13117*
**MIP-1β**	88±47	17101±5508*	18183±6645*
**RANTES**	28±9	1042±303*	909±244*
**IL-10**	ND	684±144	589±130

Media was added to skeletal myotube cultures at a concentration of 2% (diluted in regular media). *indicates significantly increased above Con, P<0.05. ND: Not detectable.

GM-CSF, granulocyte-monocyte colony-stimulating factor; IL-8, interleukin-8; TNF-α, tumor necrosis factor-α; IL-1ra, interleukin-1 receptor antagonist; MCP1, monocyte chemoattractant protein 1; MIP-1α, macrophage inflammatory protein-1α; MIP-1β, macrophage inflammatory protein-1β; RANTES, regulated upon activation normal T-cell expressed and secreted; IL-10, interleukin-10.

## Discussion

The present study demonstrates that impaired insulin sensitivity induced by cholesterol-loaded macrophages in skeletal muscle can be prevented by pre-treatment of macrophages with HDL. Further, this is demonstrated in skeletal myotubes obtained from newly diagnosed, patients with early-stage type 2 diabetes, and is therefore relevant to the potential indication of HDL-elevating agents in this population. The induction of impaired glucose uptake in skeletal muscle incubated with conditioned media from macrophages treated with acLDL and the reversal of the effect by HDL could not be attributed to cholesterol removal or a reduction in activation of common inflammatory pathways in macrophages. These observations in primary human macrophage and skeletal myotubes may offer an explanation for the increased insulin sensitivity following treatment with the HDL-raising agent, torcetrapib, a CETP inhibitor in patients with type 2 diabetes [1].

Nutrient oversupply and consequent obesity leads to metabolic dysregulation in various tissues including liver, brain, skeletal muscle, adipose tissue, pancreatic β-cells and immune cells (for review see [22]). Insulin resistance in skeletal muscle is of particular importance, since it is the primary site for post-prandial glucose disposal in humans. While defects arise that are intrinsic to this tissue as a result of nutrient oversupply, it is now well known that endocrine or paracrine signals from other dysregulated tissues can impact upon skeletal muscle to further impair insulin sensitivity [22]. In particular, excessive macrophage lipid accumulation in mice results in skeletal muscle insulin resistance [23], [24]. In addition, a study utilizing macrophage conditioned media after lipid exposure under conditions similar to those in the present study, demonstrated impaired phosphorylation of Akt in skeletal muscle [25]. Inflammatory activation of macrophages results in the release of a number of factors, the most prominent of which is TNF-α, that impair skeletal muscle insulin sensitivity [15], [26], [27]. As a result, reduction in inflammatory activation of macrophages has been considered an important target for obesity-induced insulin resistance [28], [29].

Despite recent setbacks related to clinical trials with CETP inhibitors, elevating HDL is recognised as a strategy to reduce residual cardiovascular risk in optimally managed, high-risk patients, through mechanisms which include inhibition of vascular inflammatory processes [9], [11]. It has become evident that manipulation of HDL could also provide important therapeutic value for other disease states characterized by low-grade chronic inflammation. Since low grade immune cell activation/inflammation is associated with insulin resistance in skeletal muscle, we proposed that cholesterol loading via acLDL would induce macrophage inflammation, and that addition of HDL would prevent the lipid-induced inflammatory processes that result in skeletal muscle insulin resistance.

The conditioned media model employed in this study was designed as a model of lipid loading resulting in significant free cholesterol accumulation in macrophages and subsequent induction of inflammation, as indicated by increased phosphorylation of JNK [30]. Treatment with acLDL resulted in significant cholesterol loading, increased JNK phosphorylation and release of cytokines from macrophages. Conditioned media from lipid-loaded macrophages impaired insulin-stimulated glucose uptake when transferred to human skeletal myotubes. While HDL co-treatment reversed the effect, total intracellular cholesterol content, phosphorylation of JNK and pro-inflammatory cytokine release were not altered. Furthermore, while Akt phosphorylation in these myotubes, a marker of insulin signaling, increased robustly with insulin, there was no treatment effect. This discordance between glucose disposal and insulin signaling via Akt in cultured muscle cells has been previously reported [31], supporting the emerging concept of a disconnect between the interaction of GLUT4 within the plasma membrane and classical insulin signaling [32], [33].

The factor(s) in the conditioned media accounting for the changes in insulin-mediated glucose uptake in the presence and absence of HDL thus remain unknown. Potential candidates may involve lesser known inflammatory pathways, lipids secreted into the conditioned media or reactive oxygen species. Given the important role of both LDL and HDL in cellular lipid exchange it is highly likely that the various interventions had differential effects on the lipid composition of the conditioned media. In particular co-incubation of macrophages with acLDL and HDL would be expected to result in net movement of pro-inflammatory lipids to HDL particles within the media, thus depleting the macrophages of certain lipids, and possibly enriching for others. The experimental paradigm then involved removal of this media (including lipids within the media lipoproteins) and replacement with fresh media. Macrophage lipid secretion into the conditioned media could thus be different depending on the prior presence or absence of HDL.

Given that lipids are now known to play an important role as signaling molecules [34], this is a plausible mechanism. Secreted bioactive ‘lipokines’ have been identified previously [35], and such lipids may signal directly in target cells, or alter sarcolemmal membrane dynamics, such as facilitating translocation of glucose transporters to and from the cell surface. On this basis, we conducted lipidomic analysis of the conditioned media which assessed over 230 individual lipid species using electrospray-ionisation tandem mass spectrometry (data not shown) [36]. However, none of the individual lipid species or family of lipids assessed were different in the conditioned media after the three treatments in such a way as might explain our insulin-mediated glucose uptake data.

### Limitations

A number of potential mechanisms for the observed effects were investigated in this study, but none fully explained our results. Further work is required to elucidate the exact mechanisms of the effects of pre-treatment with acLDL with and without HDL on secreted factors from macrophages regulating insulin sensitivity in skeletal muscle.

### Conclusion

While the field of HDL therapeutics has yet to deliver a safe and efficacious agent to reduce cardiovascular events, evidence is emerging for a role of HDL in glucose metabolism [1]–[3]. In the present study further weight is added to this body of literature by demonstrating that HDL restores defective insulin-stimulated glucose uptake induced by lipid laden macrophages. Additional studies are required to identify the factors responsible for mediating this process, which appear to be independent of common immunomodulatory cytokines. This study supports the concept that HDL-raising strategies may play a role in the prevention and management of type 2 diabetes.
